# Effects of Tung Oil-Based Polyols on the Thermal Stability, Flame Retardancy, and Mechanical Properties of Rigid Polyurethane Foam

**DOI:** 10.3390/polym11010045

**Published:** 2018-12-30

**Authors:** Wei Zhou, Caiying Bo, Puyou Jia, Yonghong Zhou, Meng Zhang

**Affiliations:** 1 Institute of Chemical Industry of Forestry Products, CAF, 16 Suojin North Road, Nanjing 210042, China; vvzhou90@163.com (W.Z.); newstar2002@163.com (C.B.); jiapuyou@163.com (P.J.); yhzhou777@163.com (Y.Z.); 2 Key Lab of Forest Chemical Engineering, SFA, 16 Suojin North Road, Nanjing 210042, China; 3 Key Lab of Biomass Energy and Material, Jiangsu Province, 16 Suojin North Road, Nanjing 210042, China; 4 Co-Innovation Center of Efficient Processing and Utilization of Forest Resources, Nanjing Forestry University, 159 Longpan Road, Nanjing 210037, China

**Keywords:** tung oil, DOPO, dihydroxydiphenylsiane, flame retardant, rigid polyurethane foam

## Abstract

A phosphorus-containing tung oil-based polyol (PTOP) and a silicon-containing tung oil-based polyol (PTOSi) were each efficiently prepared by attaching 9,10-dihydro-9-oxa-10-phosphaphenanthrene (DOPO) and dihydroxydiphenylsilane (DPSD) directly, respectively, to the epoxidized monoglyceride of tung oil (EGTO) through a ring-opening reaction. The two new polyols were used in the formation of rigid polyurethane foam (RPUF), which displayed great thermal stability and excellent flame retardancy performance. The limiting oxygen index (LOI) value of RPUF containing 80 wt % PTOP and 80 wt % PTOSi was 24.0% and 23.4%, respectively. Fourier transfer infrared (FTIR), Nuclear Magnetic Resonance (NMR) and thermogravimetric (TG) analysis revealed that DOPO and DPSD are linked to EGTO by a covalent bond. Interestingly, PTOP and PTOSi had opposite effects on *T*_g_ and the compressive strength of RPUF, where, with the appropriate loading, the compressive strengths were 0.82 MPa and 0.25 MPa, respectively. At a higher loading of PTOP and PTOSi, the thermal conductivity of RPUF increased while the RPUF density decreased. The scanning electron microscope (SEM) micrographs showed that the size and closed areas of the RPUF cells were regular. SEM micrographs of the char after combustion showed that the char layer was compact and dense. The enhanced flame retardancy of RPUF resulted from the barrier effect of the char layer, which was covered with incombustible substance.

## 1. Introduction

The depletion of petroleum reserves is driving the development of polymers and polymer additives from sustainable bio-materials. Polymer materials made from renewable sources have attracted attention around the world [1]. Vegetable oils are particularly promising renewable chemicals for the preparation of polyols and rigid polyurethane foam (RPUF) due to their renewability, universal availability, and environmental compatibility. Vegetable oils have double bonds, ester groups and other reactive sites that can be modified to form new polyol structures [2]. Vegetable oils such as castor oil, palm kernel oil [3,4], rapeseed oil [5], and tung oil [6] have been used to produce RPUF. RPUF has been widely used in materials for building insulation due to its excellent mechanical performance. However, a limitation to the use of RPUF is flammability, which is related to its cellular structure and low density [7,8].

In recent years, much effort has been devoted to increasing the flame-retardant properties of RPUF. Conventional methods of flame-retardant treatment for RPUF include the incorporation of flame-retardant additives based on phosphorus, silicone, nitrogen, and halogen compounds. While these flame-retardant additives impart excellent flame retardancy to RPUF, the introduction of larger amounts of flame retardants may have an adverse effect on the mechanical and physical characteristics of RPUF [9]. Furthermore, flame retardant additives may easily mobilize out of the RPUF matrix into the exterior [10]. When burning, flame retardants that contain halogens release a lot of harmful gases and bring about environmental pollution. More importantly, organohalogen compounds fragmenting from the polymers are stable and able to persist and bioaccumulate in the environment, and thus may pose a risk to human health [11]. Consequently, an environmentally friendly reactive flame retardant is needed. The main benefit of reactive flame retardants compared to other flame-retardant additives is that the flame-retardant molecule is linked to the RPUF structure with a covalent bond [12].

The compound 9,10-dihydro-9-oxa-10-phosphaphenanthrene (DOPO) [13] is an environmentally friendly reactive flame retardant that has attracted considerable attention because of its outstanding thermal stability and ability to resist oxidation and hydrolysis [14]. Due to its aromatic and phenanthrene ring structure, DOPO is more thermally and chemically stable than standard organic phosphate, and little toxic gas is released during its combustion [15]. The flame-retardant ability of DOPO has been attributed to the formation of a reactive substance such as PO^−^, which is released to the gas phase where it engages with H· and OH· groups and disrupts the combustion process [16]. DOPO exhibits prominent gas-phase flame retardant properties during RPUF combustion [17,18]. Modification of RPUF through the incorporation of silicon-containing flame retardants is also recognized as an environmentally friendly method that reduces flammability [19]. Silicone materials show good flame-retardant properties through the formation of char barriers (Si–C, Si–O) at the surface of the polymer at high temperatures [20]. There are two ways to enhance the flame retardancy of RPUF, one of which is the addition of the inorganic silicon such as nano-silica and whisker silicon oxide [21], and the other of which is the introduction of silicon into the structure of polyols using 3-aminopropylmethyldiethoxysilane (APTES), *N*-(*β*-aminoethyl)-*γ*-aminopropylmethyl dimethoxysilane (KH-602), and SiO_2_ [22,23,24]. This has resulted in the development of novel reactive flame retardants for RPUF. Tung oil-based flame-retardant polyols have not been reported previously.

The goal of this work was to develop an innovative RPUF based on the incorporation of a phosphorus-containing tung oil-based polyol (PTOP) and a silicon-containing tung oil-based polyol (PTOSi). The molecular structure of the compounds and the thermal properties of the two new tung oil-based flame-retardant polyols were characterized using FTIR, ^1^H NMR, ^31^P NMR, and TG. RPUFs were characterized for their thermal and flame-retardant behaviors using several analytical techniques.

## 2. Materials and Methods

### 2.1. Materials

Tung oil (TO) was provided by Jiangsu Qianglin Bioenergy Material Co., Ltd (Qianglin, China). Glycerol, sodium methoxide and ethyl acetate were obtained from Nanjing Chemical Reagent Co., Ltd (Nanjing, China). Acetic acid was purchased from Nanjing Chemical Reagent Co., Ltd (Nanjing, China). Hydrogen peroxide (30 wt %) was purchased from Shanghai Linfeng Chemical Reagent Co., Ltd (Shanghai, China). Other materials such as P-toluenesulfonic acid monohydrate (TsOH·H_2_O), 9,10-dihydro-9-oxa-10-phosphaphenanthrene (DOPO), dihydroxydiphenylsilane (DPSD) and dibutyltin dilaurate (DBTDL) were provided by Aladdin Industrial Corporation (Shanghai, China). Polyether polyol (PPG4110) was purchased from Jiangsu Qianglin Bioenergy Material Co., Ltd (Jiangsu, China); and its hydroxyl value was 403 mg KOH/g and its viscosity was 3.6 Pa·s at 25 °C. Polyaryl polyisocyanate (PAPI) was provided by Yantai Wanhua Polyurethane Co., Ltd (Yantai, China) with 30.3 wt % NCO. Foam stabilizer (AK8804) was purchased from Jiangsu Maysta Chemical Co., Ltd (Jiangsu, China). Foaming agent (HFC-365mfc) was obtained from Dongguan Changze Chemical Co., Ltd (Dongguan, China). Deionized water was made at the laboratory.

### 2.2. Synthesis of GTO and EGTO

**Scheme 1:**
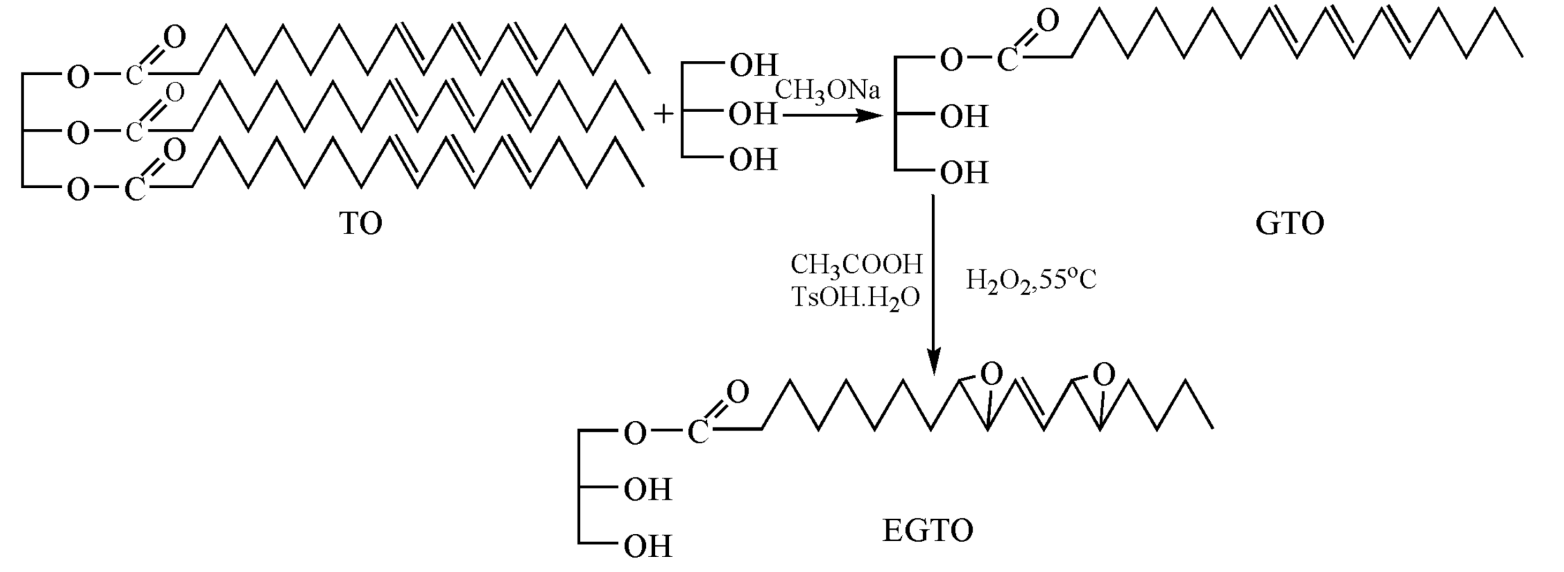
Synthesis route of tung oil monoglyceride (GTO) and epoxidized monoglyceride of tung oil (EGTO).

The synthesis of the tung oil monoglyceride (GTO) was carried out as described in a previous study [25]. The epoxidized monoglyceride of tung oil (EGTO) was prepared as follows: 100 g of GTO (0.46 mol carbon-carbon double bond), CH_3_COOH (0.92 mol) and TsOH·H_2_O (0.046 mol) were added to a round-bottom flask. Hydrogen peroxide solution (0.69 mol H_2_O_2_) was added dropwise over a period of 1 h and the reaction was allowed to proceed for 4 to 5 h at a temperature of 55 °C. The EGTO product was neutralized with 1 mol/L sodium hydrocarbonate solution and then extracted with ethyl acetate. The ethyl acetate solvent was removed by reduced pressure distillation. EGTO was obtained and its epoxy value was 3%. The synthesis of GTO and EGTO is illustrated in Scheme 1.

### 2.3. Synthesis of PTOP and PTOSi

**Scheme 2:**
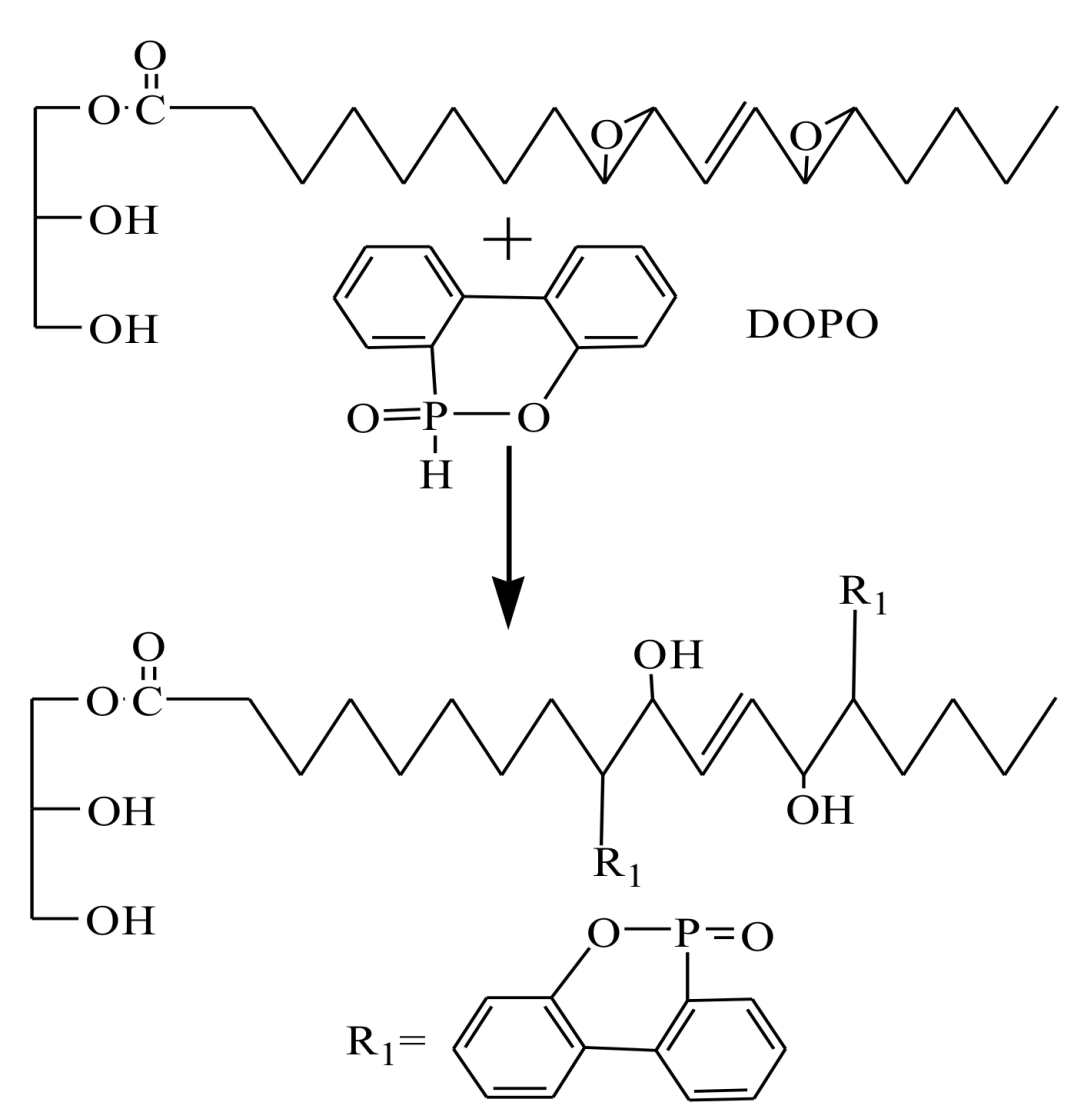
Synthesis route of phosphorus-containing tung oil-based polyol (PTOP).

**Scheme 3:**
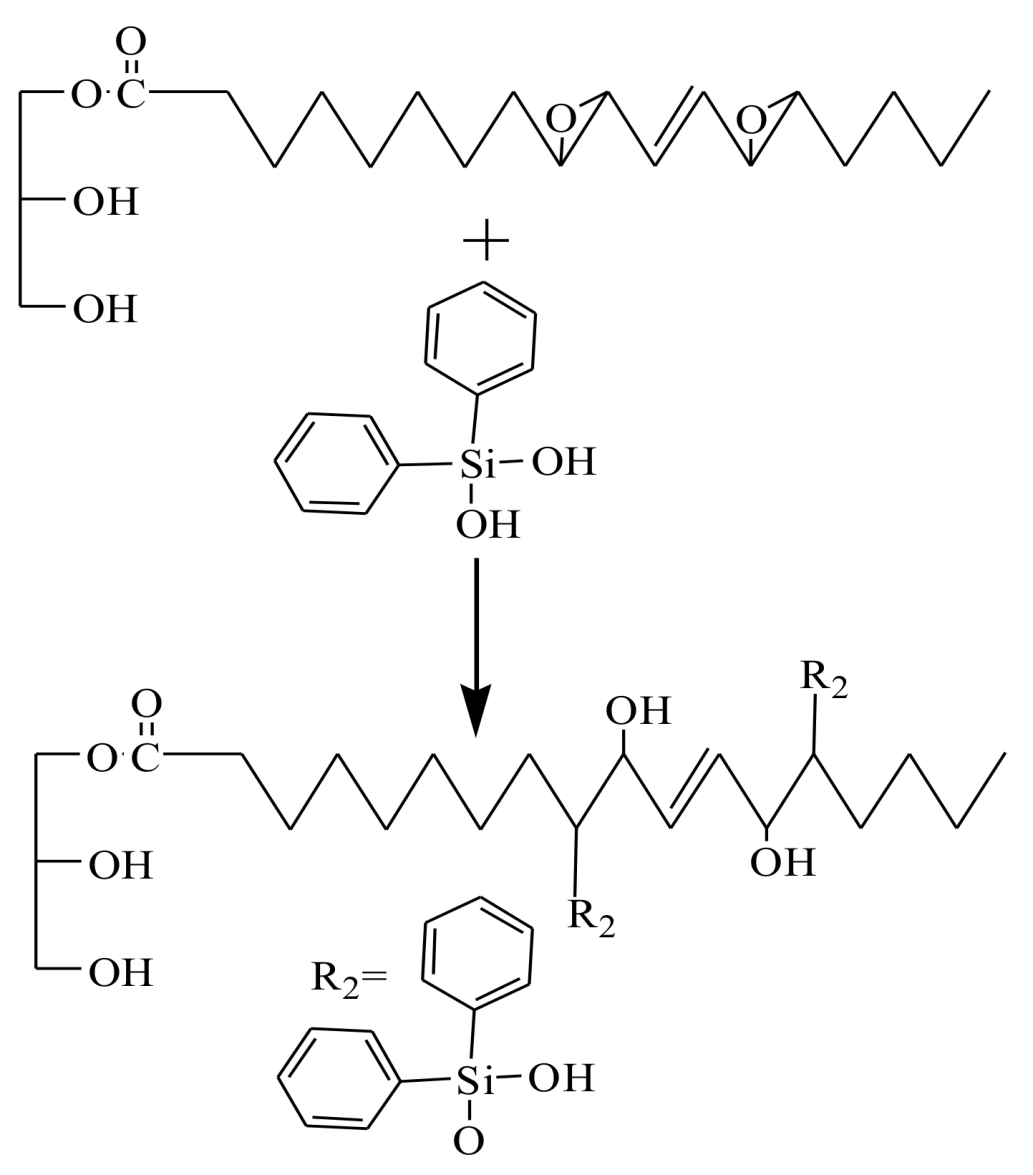
Synthesis route of silicon-containing tung oil-based polyol (PTOSi).

The reaction equations for PTOP and PTOSi are shown in Scheme 2 and Scheme 3 and the parameters for PTOP and PTOSi are summarized in Table 1. The reaction temperature and time of PTOP were 130 °C and 120 min, respectively. After the reaction, the product was allowed to cool to room temperature and to stand for 48 h, after which a small amount of unreacted DOPO precipitated as a white solid. The brown clear transparent liquid at the top was washed to neutral with distilled water, which was distilled by vacuum distillation. The conversion rate of DOPO was obtained by comparing the change in the epoxy value of PTOP with that of EGTO. EGTO and DPSD were blended and heated to 140 °C for 2 h. After the product was cooled down to room temperature and allowed to stand for 48 h, a small amount of the white solid of the unreacted DPSD subsided, the top brown clear transparent liquid was washed to neutral with distilled water and water was removed by reduced pressure distillation. The conversion rate of PTOSi was obtained by comparing the change in the epoxy value of PTOSi with that of EGTO. 

### 2.4. Preparation of RPUF

RPUF was prepared using a one-step method. The raw materials and formulations used in the preparation of RPUF are listed in Table 2. Firstly, Part A was made up of AK8804, DBTDL, blowing agent, water, PPG4110 and flame-retardant polyols. Then, the mixture of PAPI (Part B) and Part A was stirred for 2 min at approximately 2000–2500 rpm to ensure homogeneous mixing. At the end, the mixture was poured into a mold and allowed to cure at 80 °C for 24 h. The P content of blended polyols of RPUF/P60 and RPUF/P80 were 1.11% and 1.48%, respectively, and the Si content of blended polyols of RPUF/Si60 and RPUF/Si80 were 1.14% and 1.52%, respectively.

### 2.5. Characterization

#### 2.5.1. Physical Parameter Analysis

The hydroxyl value and acid value of the sample were determined according to ASTM S957-86 and ASTM D4662-03, respectively. The epoxy value of the sample was tested according to GB/T 1677-2008. The viscosity of the sample was determined based on ASTM D2983 using a rotary Viscometer DVS+ (Brookfield, Middleboro, MA, USA).

#### 2.5.2. Gel Permeation Chromatography (GPC)

The reaction progress of PTOP was tracked by GPC (Waters, Milford, MA, USA). GPC spectra were obtained at a temperature of 30 °C and flow rate of 1 mL/min. The parameters of the column were as follows: mixed PL gel 300 × 718 mm, 25 µm. The solvent was THF.

#### 2.5.3. Fourier Transform Infrared (FTIR)

FTIR spectra of each specimen was measured using a Nicolet iS10 FTIR spectrometer (Madison, WI, USA). The wavenumber range was from 500 to 4000 cm^−1^ at a resolution of 4 cm^−1^.

#### 2.5.4. Nuclear Magnetic Resonance (NMR)

Nuclear magnetic resonance (^1^H NMR) spectra and ^31^PNMR spectra were measured using a BRUKER AV-300 (300MHz) (Bruker BioSpin AG Facilities, Fallanden, Switzerland). Deuterated chloroform was used as the solvent and tetramethylsilane (TMS) and H_3_PO_4_ were used as internal standards.

#### 2.5.5. Scanning Electron Microscope (SEM)

The microstructure of the RPUF and RPUF residue was determined by SEM (3400N, Hitachi, Tokyo, Japan).

#### 2.5.6. Thermogravimetric Analysis (TGA)

The thermal stability of the specimen was tested using a NETZSCH TG 209F3 (Gebruder-Netzsch-Straße, Germany). Each specimen was heated from 40 to 800 °C under an N2 atmosphere with a flow rate of 20 mL/min and a heating rate of 10 °C/min in alumina crucible.

#### 2.5.7. Differential Scanning Calorimeter (DSC)

The thermal property of RPUF was analyzed by DSC on a NETZSCH DSC 200F3 (Gebruder-Netzsch-Straße, Germany). Samples of about 10 mg were weighed and sealed in the aluminum DSC pans and placed in the DSC cell. They were first cooled for 0.5 min at −60 °C, and then heated from −60 to 200 °C at the rate of 20 °C/min under nitrogen atmosphere. They were kept at 200 °C for 0.5 min to eliminate the previous heat history and subsequently cooled to −60 °C at 20 °C/min. Lastly, they were heated again to 200 °C at 20 °C/min.

#### 2.5.8. Limit Oxygen Index (LOI) Test 

The limiting oxygen index test was performed according to ASTM D4986 and using an LOI Meter (PX-01-005, Nanjing Analytical Instrument Factory Co., Ltd, Nanjing, China). The dimensions of the specimen were 100 × 10 × 10 mm^3^. Each sample was run twice and the average result was calculated.

#### 2.5.9. Cone Calorimeter Test (CTT)

CTT was carried out by employing a cone calorimeter (FTT2000, Fire Testing Technology, West Sussex, UK) according to ISO5660-1. The dimensions of the sample were 20 × 100 × 100 mm^3^, which was enveloped in aluminum foil. FTT2000 provided the external heat flux at 35 kW/m^2^ horizontally. Each specimen was detected two times and the average result was obtained.

#### 2.5.10. Compressive Strength Test

The compressive strength of RPUF was measured according to ASTM D1621. It was measured using a CMT4000 universal testing machine (Shenzhen Suns Technology Stock Co., Ltd, Shenzhen, China) where a speed of 2 mm/s of compressive force was adopted and each sample was tested 5 times and the average result was obtained.

#### 2.5.11. Thermal Conductivity Test

The thermal conductivity of RPUF was examined using a thermal conductivity analyzer (JB-DZDR-P, Shanghai Jiubin Instrument Co., Ltd, Shanghai, China) according to ASTM C518. The precision of the sample was 50 × 50 × 20 mm^3^.

#### 2.5.12. Density Test

The density of RPUF was measured according to ASTM D1622. The measurement of the sample was 50 × 50 × 50 mm^3^, and the density value was obtained from the average of five repeated tests.

## 3. Results

### 3.1. Synthesis of PTOP

**Figure 1:**
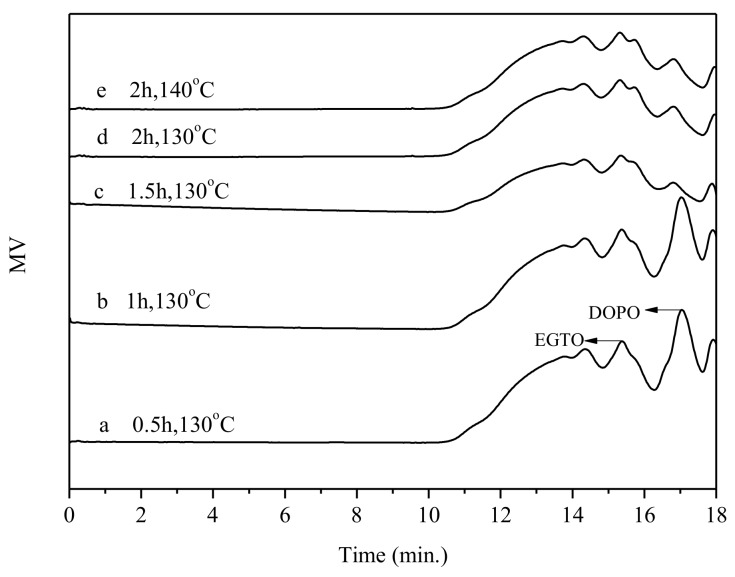
The Gel Permeation Chromatography (GPC) curves of time.

The reaction progress of PTOP was monitored by GPC at different times. In Figure 1a, the peak at 17.25 min was attributed to DOPO, and the broad peak from 10 min to 15.40 min was assigned to PTOP, while the narrow peak from 15.40 min to 16.50 min was assigned to EGTO. Due to the excessive amount of EGTO, the peak representing EGTO was evident during the PTOP reaction process. The DOPO peak almost disappeared at 2 h, which implies that the reaction between DOPO and EGTO was completed. It can be seen from the Figure 1e,d that no DOPO remained after 2 h at 130 °C and 140 °C. High temperatures may cause other side reactions to occur. Thus, the synthesis of PTOP was conducted for 2 h at a temperature below 130 °C.

### 3.2. FTIR of EGTO, PTOP, and PTOSi

**Figure 2:**
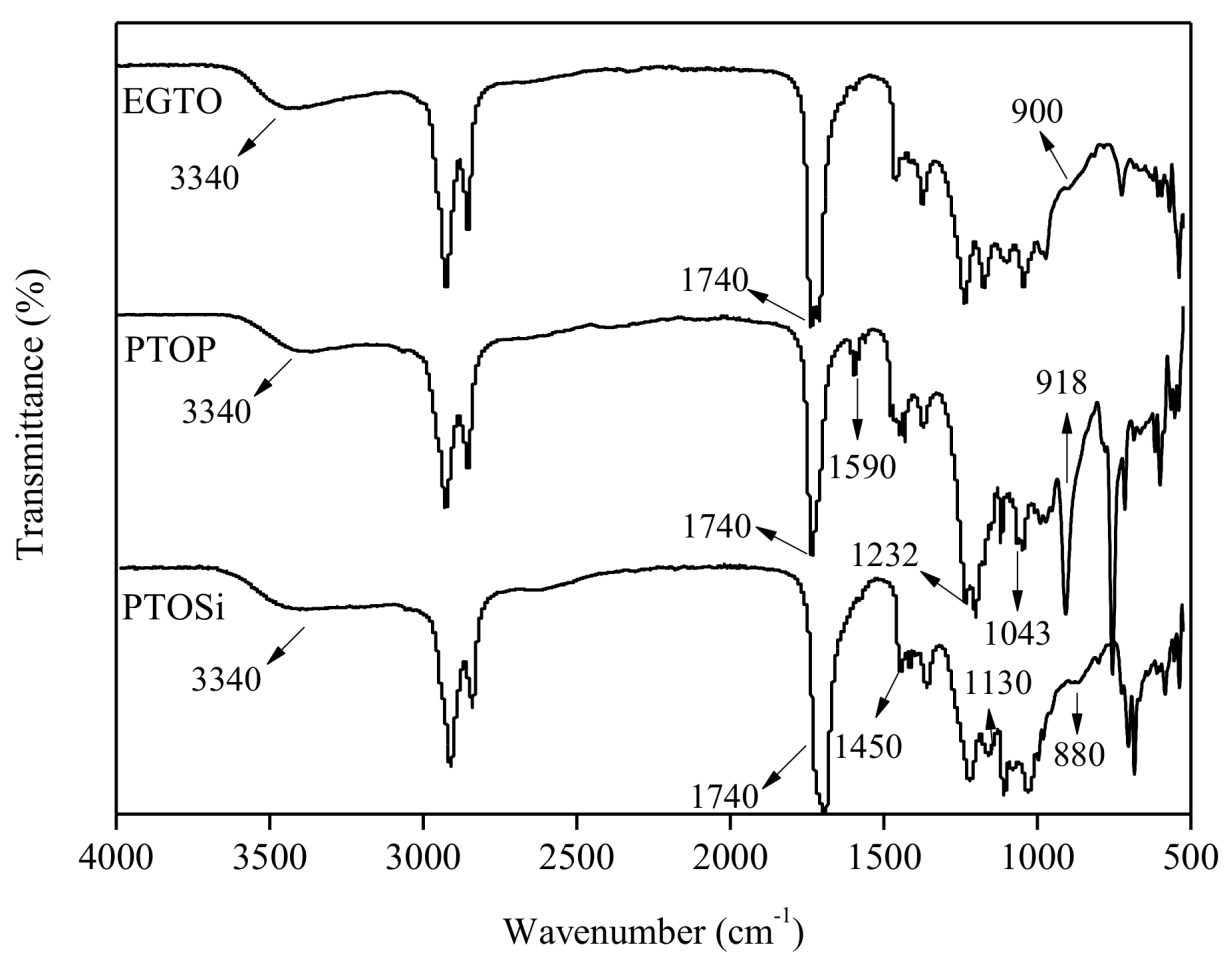
Fourier transfer infrared (FTIR) spectra of EGTO, PTOP, and PTOSi.

Figure 2 presents the FTIR spectra of EGTO, PTOP, and PTOSi. Hydroxyl absorption in EGTO appeared at 3440 cm^−1^, while the –CH_2_– stretching vibration peaks appeared at 2930 and 2850 cm^−1^. The stretching vibration at 900 cm^−1^ belonged to the epoxy group in EGTO [26]. The characteristic peak for P(O)–H stretching of DOPO in PTOP at 2428 cm^−1^ disappeared, while a broad peak appeared at 3340 cm^−1^, indicating that the epoxy ring in EGTO opened and a hydroxyl group was formed. Simultaneously, the characteristic –OH peak in PTOP occurred around 3440 cm^−1^, while the absorption peak at 1590 cm^−1^ was attributed to P–Ph, and the absorbing peaks of P=O appeared at 1232 and 1198 cm^−1^. The absorption peaks around 1043 and 918 cm^−1^ corresponded to P–O–C and P–O–Ph vibrations, which are characteristics of EGTO and DOPO, respectively [27]. The absorption peaks on the FTIR spectrum of PTOSi were consistent with observations from the literature [28]. The presence of a pronounced peak at 1450 cm^−1^ characteristic of benzene was significant. Peaks at 1070–1130 cm^−1^ corresponded to C–O and Si–O bonding [22], and the stretching vibration of two peaks at 880 and 730 cm^−1^ were assigned to the Si–C and C–H bonds of benzene. All the above changes confirmed that PTOP and PTOSi were formed via the ring opening reaction.

### 3.3. ^1^H NMR and ^31^P NMR

**Figure 3:**
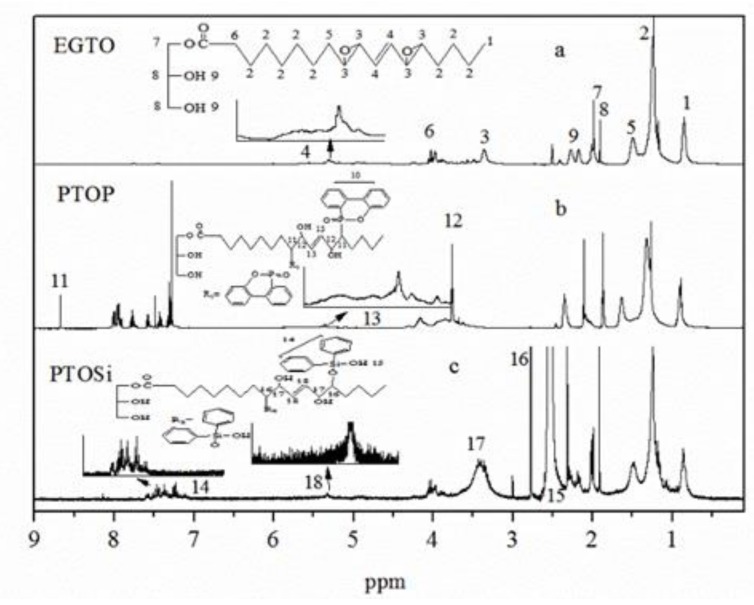
The ^1^HNMR spectra of EGTO, PTOP, and PTOSi.

**Figure 4:**
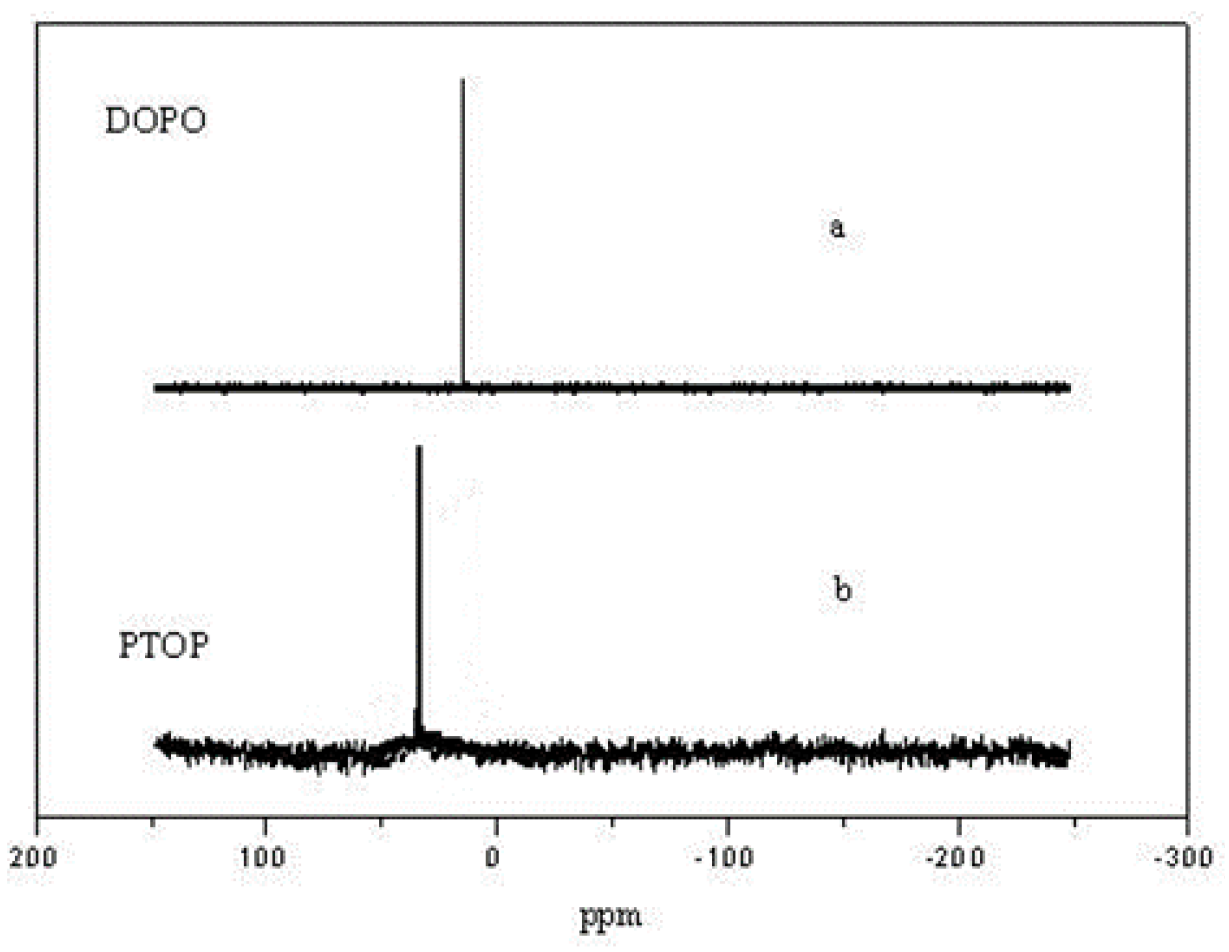
The ^31^P NMR of DOPO and PTOP.

Figure 3a presents peaks at 0.9 ppm and 2.5 ppm, which were attributed to methyl protons (peak 1) and fatty acid protons [(–CH_2_) –CO–] (peak 5), respectively. While protons in –CH_2_– (peak 6) from glycerol appeared from 4.0 to 4.1 ppm, the peak at 5.3 to 5.4 ppm was attributed to the carbon–carbon double bond. The peak at 3.3 ppm in the EGTO spectra indicates that the epoxy group (peak 3) was formed after epoxidation. The peak at 1.9 ppm corresponded to the hydrogen (peak 8) attached to the carbon adjacent to –OH and the peak that appeared at 2.0 ppm represented the hydrogen atoms adjacent to the ester bond [–O–CO–] (peak 7). In Figure 3b, the chemical shifts at 7.2–8.2 ppm (peak 10) indicated the Ar–H of PTOP, while the absorption peaks at 8.65 ppm (peak 11) and 3.60 ppm (peak 12) represented the P–C–H and –OH of PTOP, respectively. All of these characteristic ^1^H NMR bands matched the PTOP structure. In Figure 3c, the peaks at 7.2–8.2 ppm (peak 14) were associated with the Ar–H of PTOSi, while the peak at 3.4 ppm (peak 17) was characteristic of the –OH of DPSD that formed from the epoxy group via a ring opening reaction. The peak at 2.8 ppm (peak 16) was assigned to Si–O–CH–, while the –Si–OH peak appeared at 2.66 ppm (peak 15). Furthermore, PTOP exhibits a single peak at 35.50 ppm while there is no peak at 14.90 ppm in the ^31^PNMR spectrum (Figure 4). The chemical shift at 14.90 ppm was attributed to DOPO, which reveals that the reaction between DOPO and EGTO was completed. 

### 3.4. SEM Micrographs of RPUF

**Figure 5:**
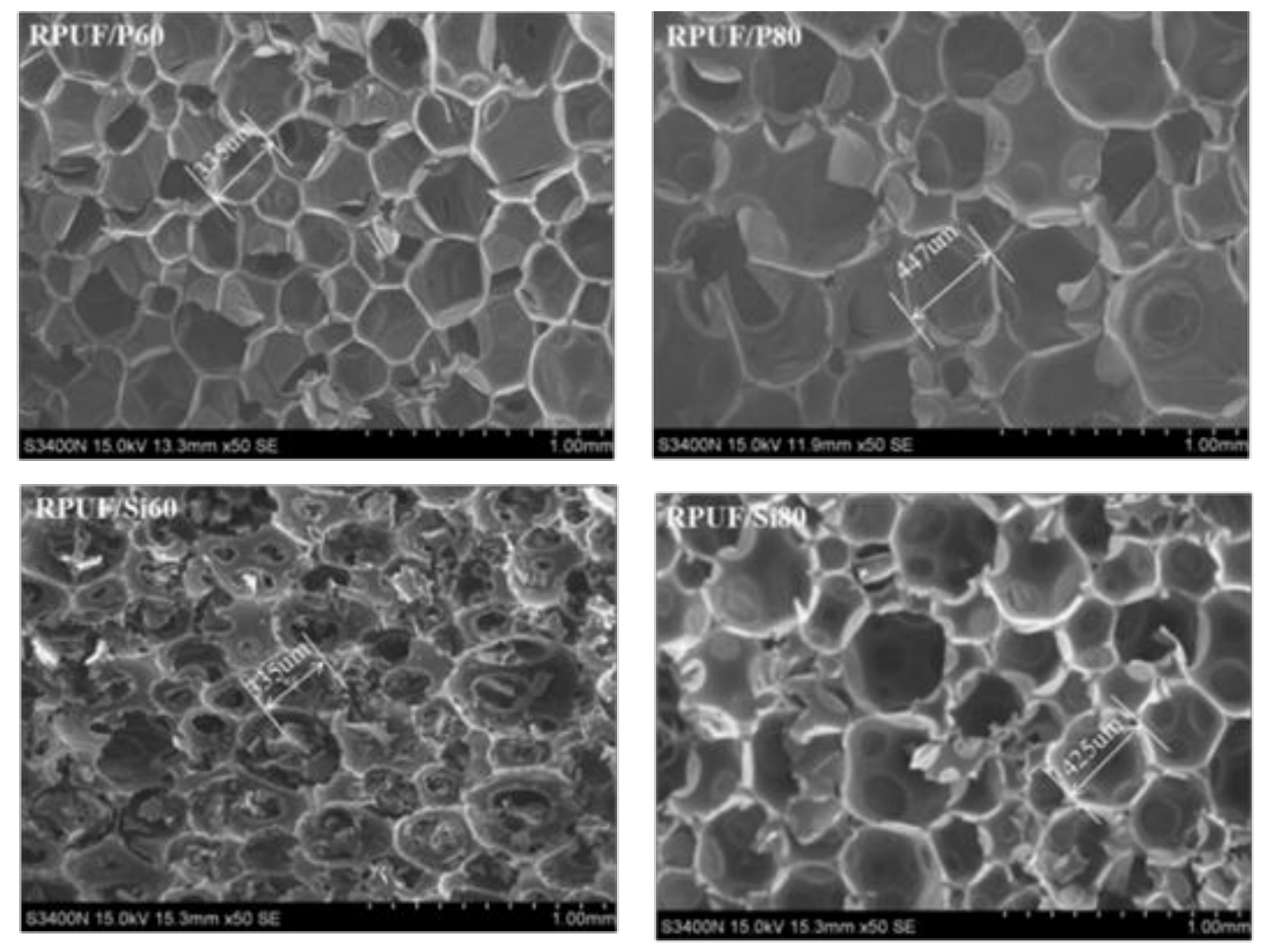
SEM of rigid polyurethane foam (RPUF).

The SEM micrographs of RPUF are presented in Figure 5, where the main structural areas noted are the cells, cell walls, and wall joints [29]. The size range of the cells increased with an increase in PTOP content. This phenomenon was also observed for RPUF/Si60 and RPUF/Si80 with an increase in PTOSi content. It was noted that a number of foam cell orifices and the size of regular cells in RPUF/P60 were consistent with those in RPUF/Si60. However, for RPUF/P80 and RPUF/Si80, the number of cell windows is decreased. The insufficient cross-linking action of PTOP and PTOSi with low functionality leads to a decrease in the number of closed foam cells, and the collapse of the newly formed foam structure due to the decrease in the number of closed foam cells.

### 3.5. Thermal Property of PTOP, PTOSi, and RPUF

#### 3.5.1. Thermal stability of PTOP, PTOSi, and RPUF

**Figure 6:**
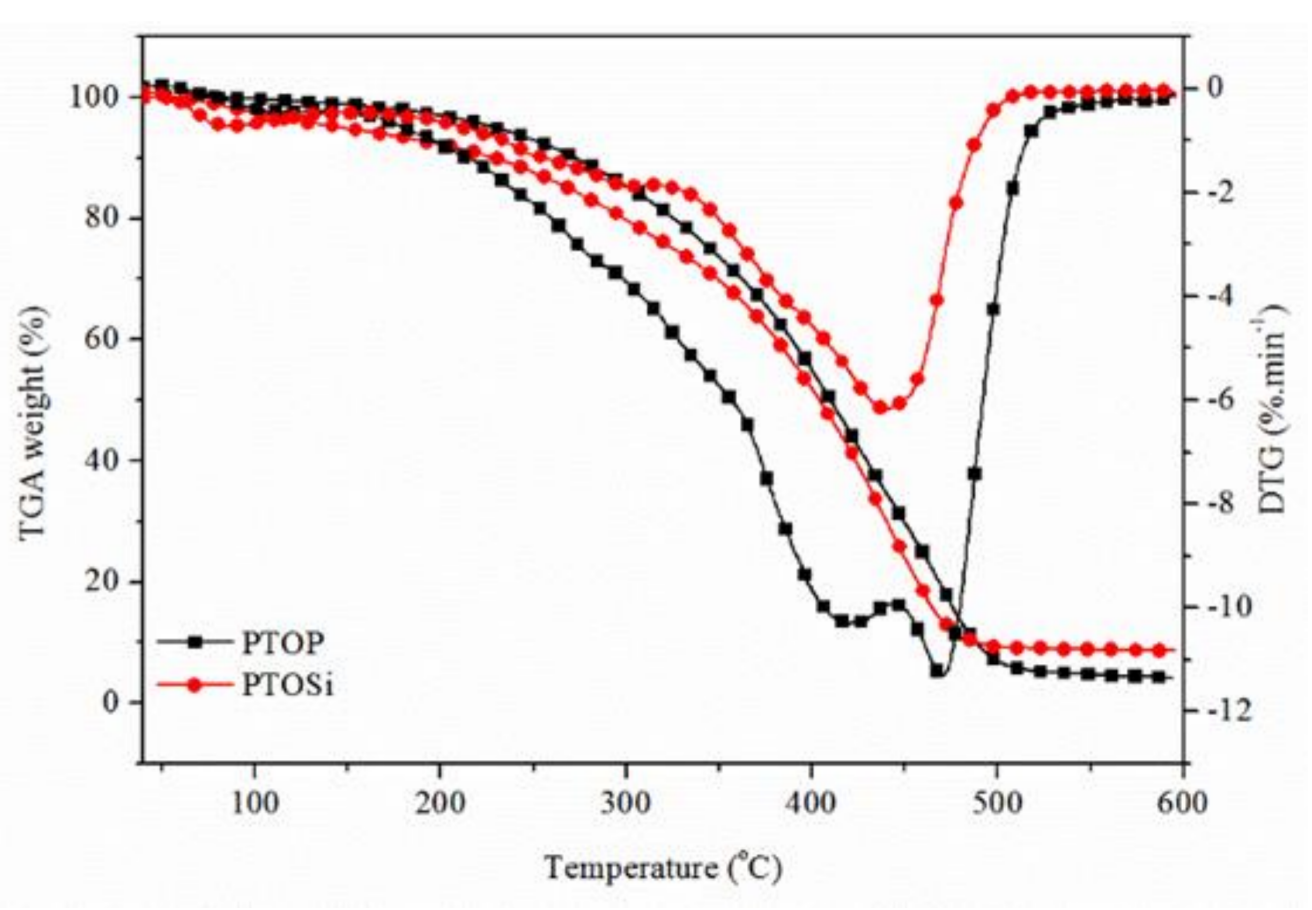
Thermogravimetric (TG) and DTG curves for PTOP and PTOSi.

**Figure 7:**
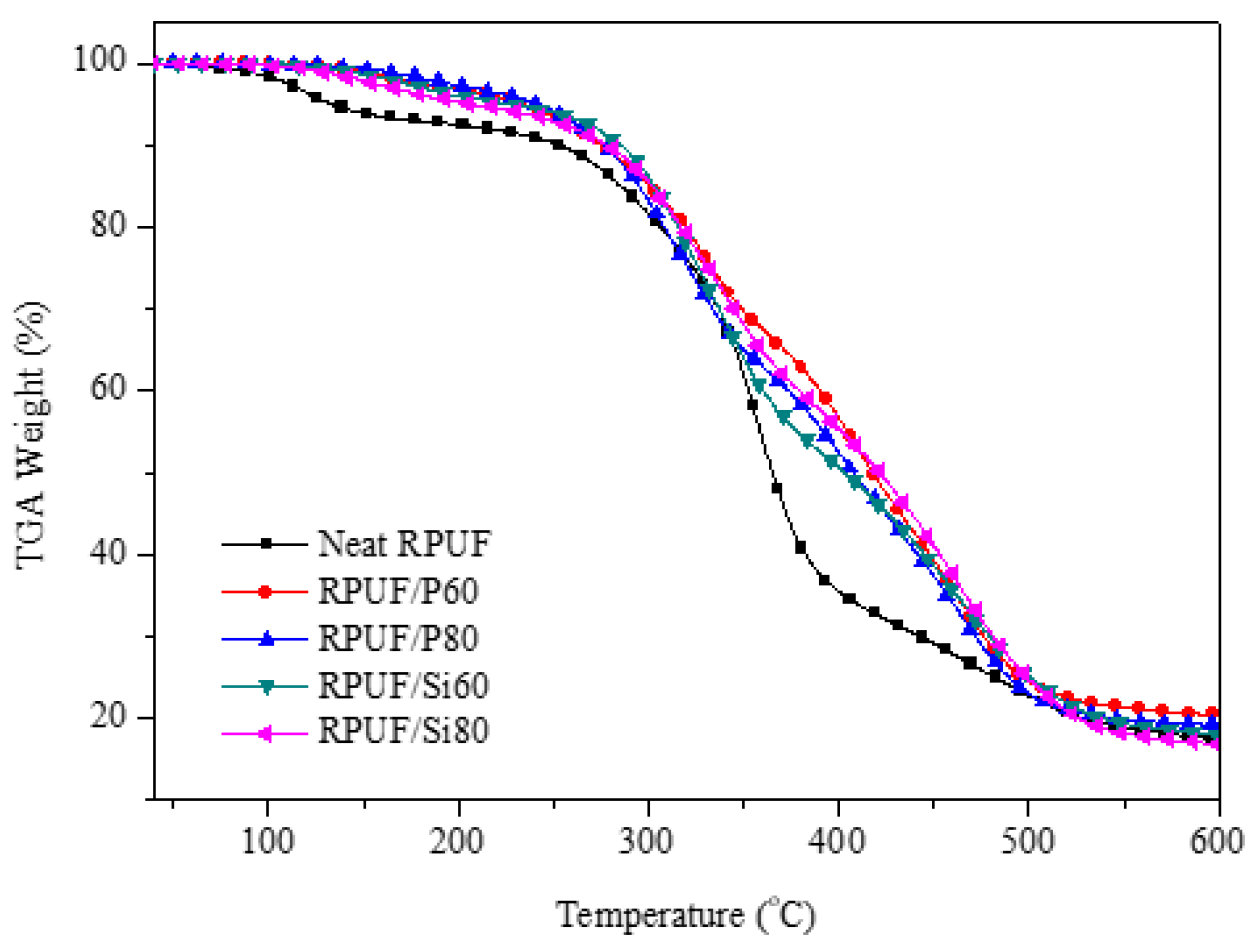
TG curves for RPUF.

**Figure 8:**
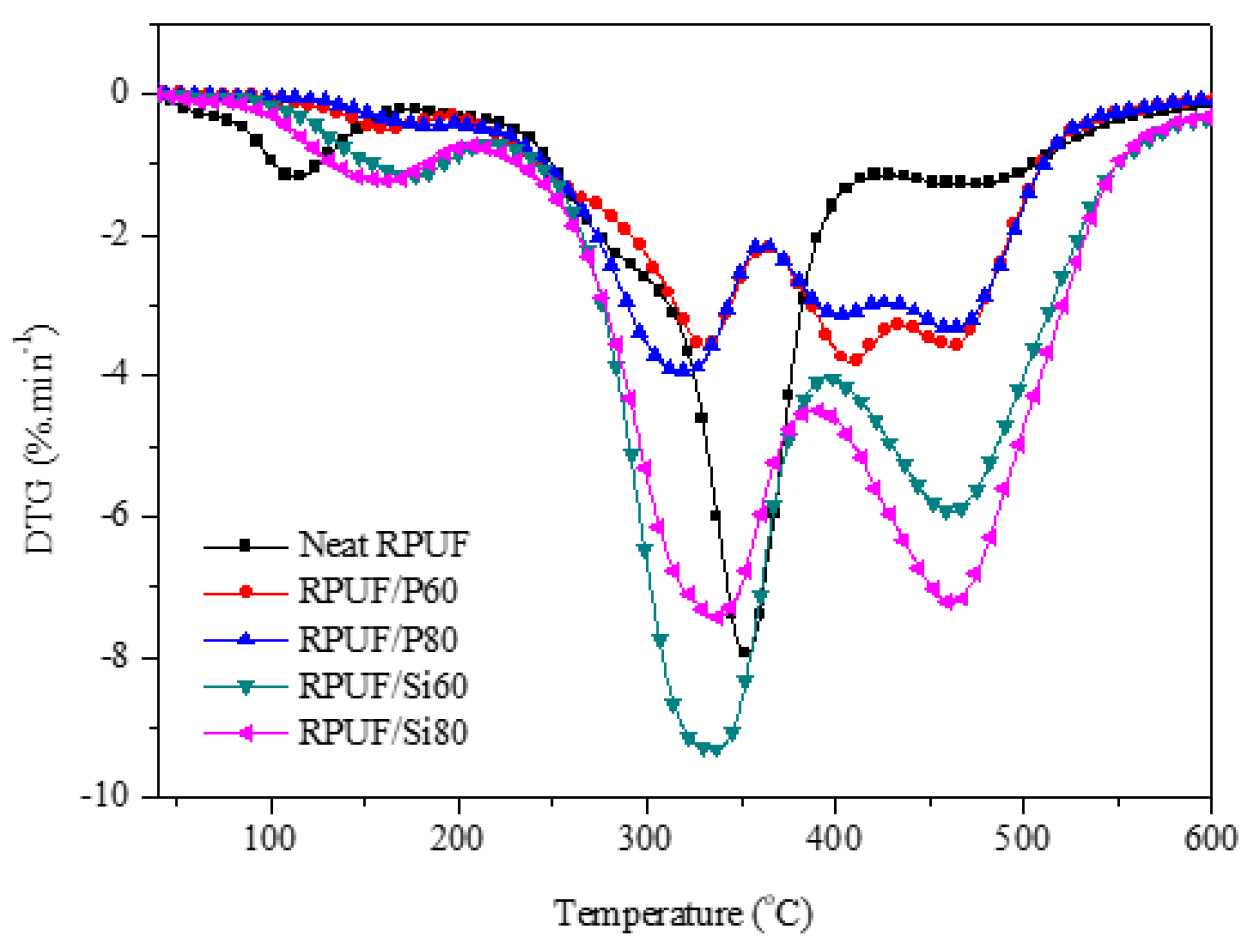
DTG curves for RPUF.

Figure 6, Figure 7 and Figure 8 show the TG and DTG curves of PTOP, PTOSi, and RPUF, respectively. *T*_onset_ represents the temperature at 10% weight loss, *T*_max_ represents the temperature when the weight loss rate was maximum, and the residue rate was obtained at 600 °C. These parameters are summarized in Table 3 and Table 4.

As can be seen from Figure 6, PTOP exhibited a higher *T*_onset_ than PTOSi, which was attributed to the low temperature of the initial DPSD degradation. Moreover, its *T*_max_ was higher than that of PTOSi, while the residue yield of PTOP was less than that of PTOSi. Silicon retained in the chemical bond could be acted as a barrier that prevents the transfer of heat and mass in the condensed phase [30]. These results indicate that the thermal property of PTOP is higher than that of PTOSi.

Thermal degradation of RPUF/P60 and RPUF/P80 showed four stages at 100 to 200 °C, 200 to 350 °C, 350 to 450 °C, and 450 to 600 °C, respectively. The first stage was attributed to the entrapment of volatile compounds in closed cells, while the other stages were attributed to the hard and soft segments of polyurethane. With an increase in the PTOP content of RPUF, the residue rate at 600 °C after complete decomposition becomes smaller, indicating that the degradation products of DOPO have mainly decomposed to form volatile products. The initial decomposition temperature of RPUF/P80 was lower than that of RPUF/P60, and the decreased *T*_onset_ could be attributed to the prior chemical decomposition of DOPO compared to PPG4110. Step I of *T*_max_ was associated with the urethane unit, which was considered as the hard segment in the polyurethane network. Step II of *T*_max_ was attributed to the soft segments, which were mainly made up of long chains of PPG4110 and PTOP. While step III of *T*_max_ was affected by the aromatic components in RPUF, the aromatic components were released within the temperature range of 400 to 600°C [31]. The condensation, chain scission, and cross-linking reactions of PTOP and the decomposition mechanism of PTOP in RPUF were summarized in that acid source, and polyphosphate compound could be produced via degradation in the step III.

Two stages showed in the degradation process of neat RPUF, RPUF/Si60, and RPUF/Si80. The initial thermal temperature and *T*_max_ of neat RPUF were highest among the five samples, due to the hydroxyl functionality of PPG4110 was higher than those for PTOP and PTOSi, the relative molecular weight and the cross-linking yield of neat RPUF were highest. While the maximum decomposition rate was higher than these for other samples, results reveal that the neat RPUF was degraded quickly when the temperature was higher than the initial thermal temperature.

The initial thermal decomposition and residue rates of RPUF/Si60 and RPUF/Si80 were similar to those for RPUF/P60 and RPUF/P80, while the change in following characteristic parameters such as *T*_max_ were contrary. The degradation of RPUF/Si60 and RPUF/Si80 was divided into two steps. The original degradation was ascribed to the urethane unit in the cross-linking network. Substances containing benzene structures and Si-containing groups in the RPUF were degraded when the temperature was raised.

#### 3.5.2. DSC of RPUF

**Figure 9:**
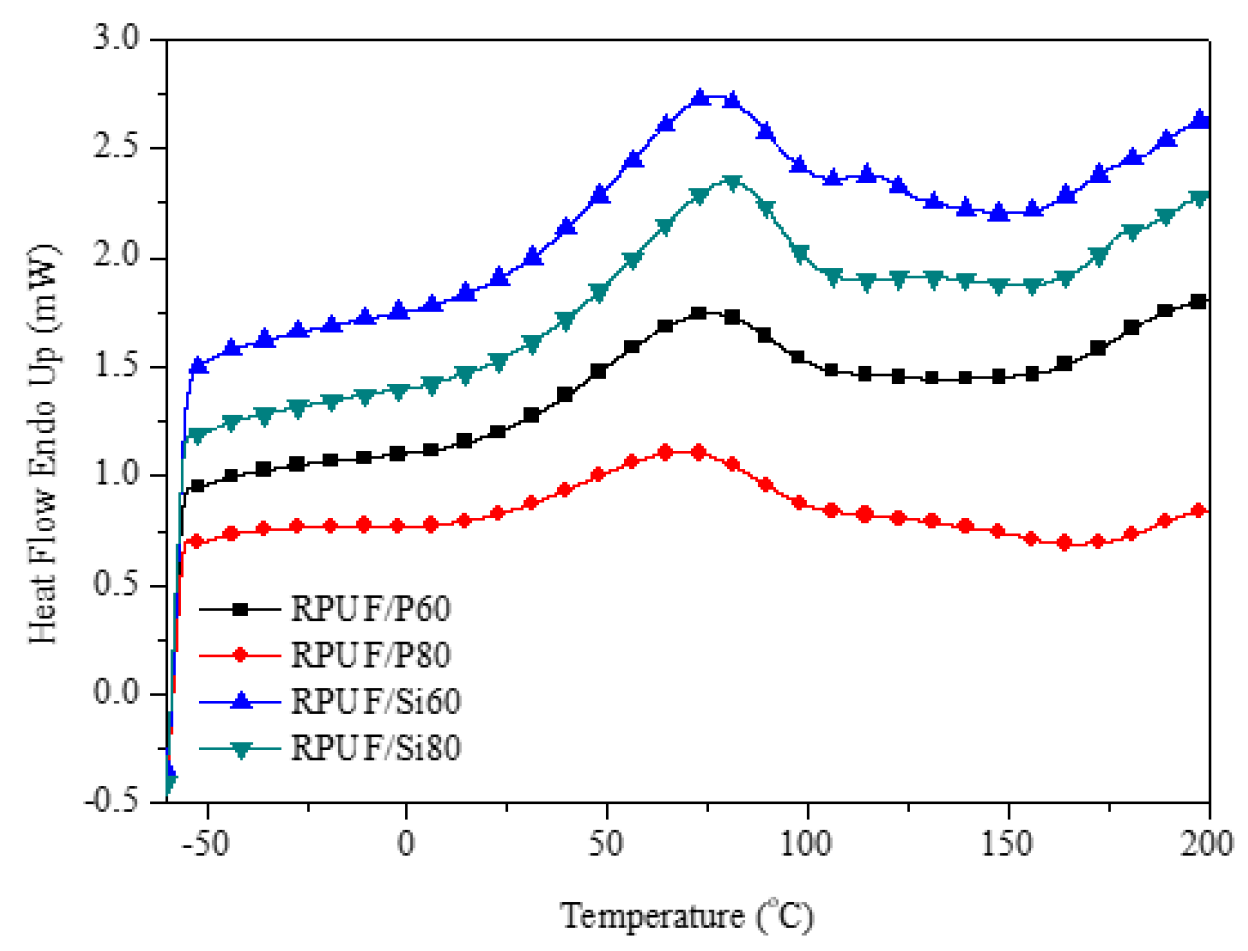
Differential Scanning Calorimetry (DSC) of RPUF.

Thermal property of RPUF was also studied by DSC, the corresponding heating thermograms are shown in Figure 9. The molecular chain of polyurethane foam was generally composed of the soft segment and hard segment. At the room temperature, soft segment was in an elastomeric state, which has an effect on low temperature performance, flexibility, organic solvent resistance and the weather-ability on RPUF. While the hard segment was in a glassy or crystalline state, which affects the melting point, thermal stability, hardness and modulus of RPUF. The temperature range of glassy transition of RPUF was from 29 to 102 °C, the *T*_g_ of RPUF/P60, RPUF/P80, RPUF/Si60, and RPUF/Si80 were 70.72, 65.08, 73.17, and 77.95 °C, respectively. The soft segment was made up of the tung oil-based flame retardant polyols and PPG4110, the hard segment was made up of isocyanate. The reason why the *T*_g_ of RPUF was less when increasing the amount of PTOP was that PTOP could be acted as the soft segment. On the contrary, with increasing the amount of PTOSi, the *T*_g_ of RPUF was higher due to PTOSi could be acted as soft segment.

### 3.6. Flame Retardancy

#### 3.6.1. Limiting Oxygen Index (LOI) Test of RPUF

Air usually contains almost 21% O_2_, so materials with an LOI value of 21% or less will burn quickly in air [6]. With the accession of PTOP and PTOSi, the LOI value of RPUF increased, indicating that both of these are flame retardant polyols. The LOI values for RPUF are listed in Table 5, where it can be seen that they are higher than that of neat RPUF, which is 19.0%. With increasing amounts of PTOP and PTOSi, the LOI value increased from 21.3% for RPUF/P60 to 24.0% for RPUF/P80, while a lower increase in the LOI value was observed for RPUF/Si60 and RPUF/Si80 from 21.0% to 23.4%. This result was attributed to the large number of PO^−^, PO_2_^−^, and P_2_O_4_^−^. Free radicals that were generated and eliminated the combustible gas during the gaseous phase. Meanwhile, phosphate and its derivatives were formed from PO^−^, PO_2_^−^, and P_2_O_4_^−^, and served as a barrier to control the transfer of heat and combustible gas during the burning process [24]. During the combustion of RPUF/Si60 and RPUF/Si80, silicones can effectively shield via silica residue left when under oxygen at elevated temperatures [32]. Silica plays the role of an “insulating blanket” as a barrier to delay volatile mass transport during the decomposition. In Table 5, the LOI value of RPUF. Based on the LOI value of RPUF, PTOP showed better flame-retardant performance than that of PTOSi.

#### 3.6.2. Fire Behaviors of RPUF 

The heat release rate (HRR), peak of heat release rate (PHRR), time to ignition (TII), and total heat released (THR) measured for RPUF during the cone calorimetry test are presented in Table 6.

**Figure 10:**
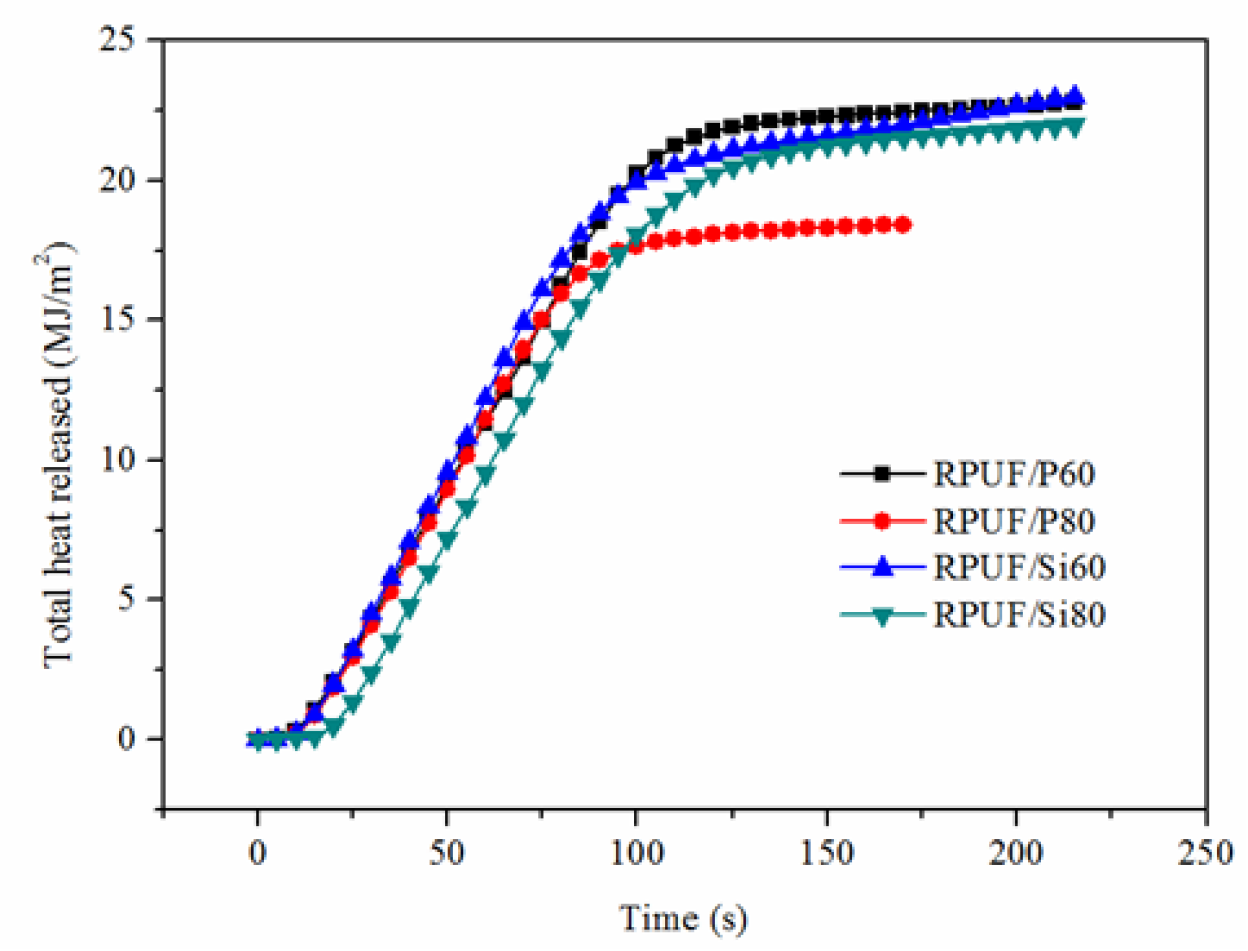
Heat release rate (HRR) curves of RPUF.

Very similar HRR curves are obtained for RPUF in Figure 10 when the PTOP and PTOSi content is increased, where the curves are seen to decrease but then quickly increase to attain the second HRR peak. RPUF ignited within the first few seconds (about 2–3 s) of exposure to heat burned steadily for 175 to 230 s. The first sharp peaks for RPUF were turned up at 35, 40, 30, and 40 s, respectively. Then, narrow peaks for each RPUF appeared at 75, 60, 60, and 65 s. The protective char layer formed from the first peak in the HRR curves, then the protective char layer was degraded, which is represented by the second peak was [33]. The HRR peaks for RPUF/P80 and RPUF/Si80 were slightly smaller and thinner, while the peak times were lengthened with an increase in the amount of PTOP and PTOSi. This may be attributed to the introduction of PTOP and PTOSi into the soft segments, contributing to the formation of the protective and continuous char layer.

**Figure 11:**
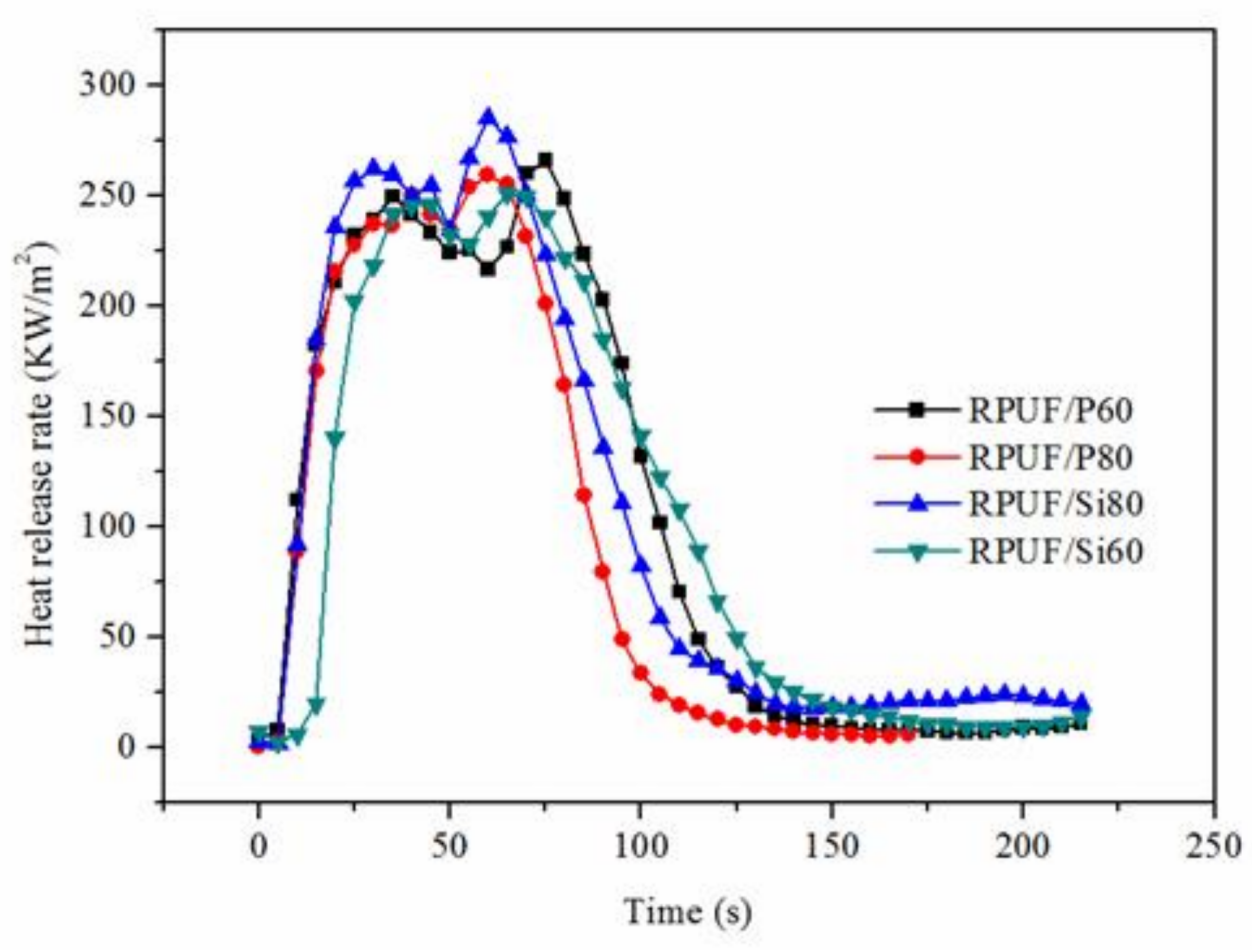
Total heat released (THR) curves of RPUF.

THR curves in Figure 11 illustrate that the value of THR decreases in the order of RPUF/P60>RPUF/Si60>RPUF/Si80>RPUF/P80. For example, the THR of RPUF/P80 is 18.40 MJ·m^−2^, a 19.30% reduction compared to that of RPUF/P60. There are two reasons for this result. On the one hand, heat is taken away by the condensed-phase and gas-phase actions of the volatile aromatic phosphates in RPUF/P60 and RPUF/P80. On the other hand, the barrier layer produced by PTOP and PTOSi can prevent heat, oxygen and flammable substance from transferring. Moreover, non-flammable gas in the gas phase system can also effectively reduce the heat released from RPUF/P80 [34]. In comparing to RPUF/Si60, RPUF/Si80 has the preferable flame-retardant property indicated by both the LOI value and CTT. The excellent flame-retardant behavior of PTOSi was illustrated by its better dispersion within the RPUF and movement towards the RPUF surface during the burning process. A highly flame-retardant char generated from the combustion of PTOSi can reduce the total heat released. 

Based on related parameters such as PHRR and THR presented in Table 6, the flame-retardant efficiency of PTOP is higher than that of PTOSi. This result accounts for the fact that PTOP can migrate to the surface of the RPUF during combustion and allow for the formation of the condensed-phase and gas-phase of volatile aromatic phosphates. This indicates that the addition of PTOP and PTOSi can boost the fire-inhibition activity during combustion.

#### 3.6.3. SEM and EDAX of RPUF Residue 

**Figure 12:**
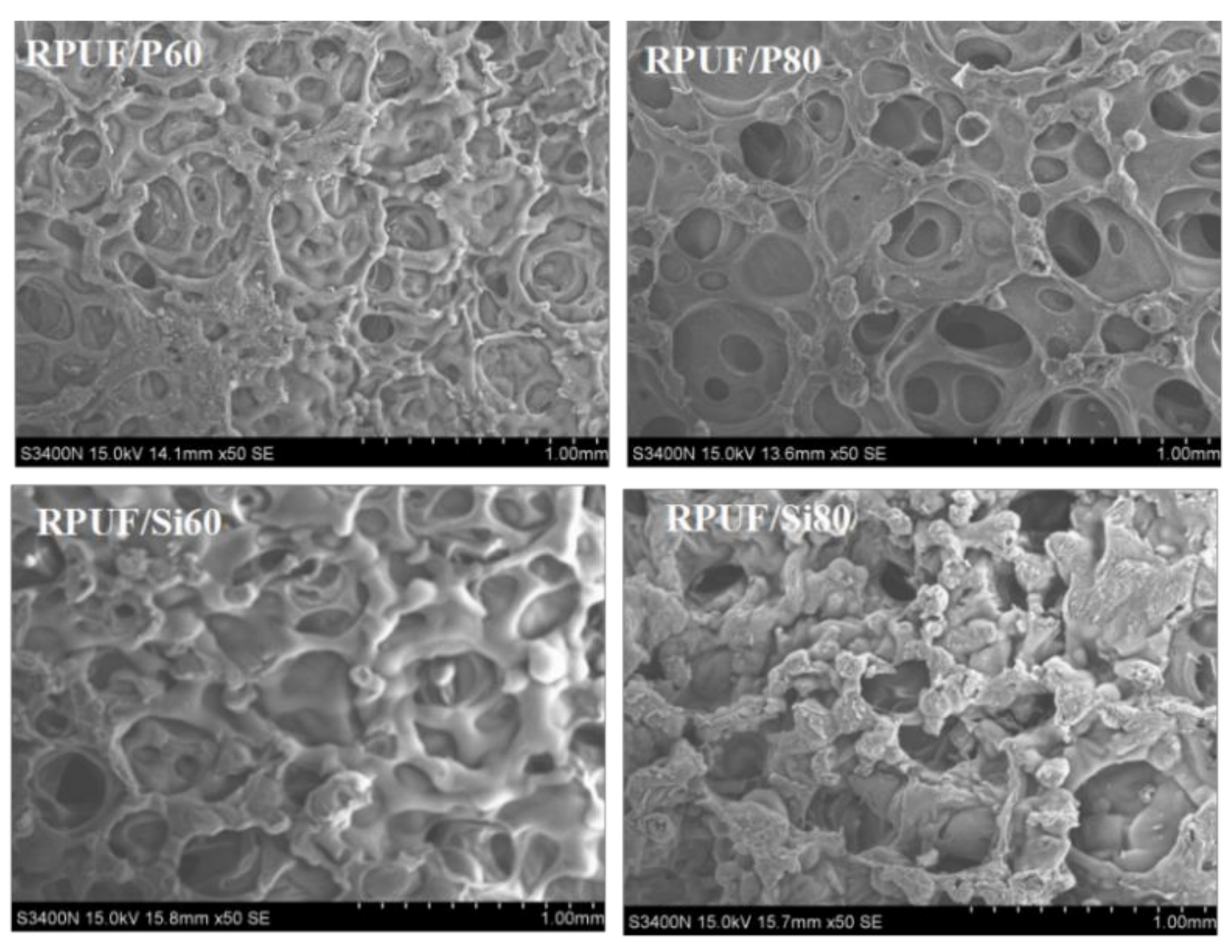
SEM of RPUF residue.

To confirm that P-containing compounds and Si-containing compounds were retained in the residue and acted as the flame retardant barrier. The microstructure of the residue after combustion was examined by SEM, RPUF/P60, RPUF/P80, RPUF/Si60, and RPUF/Si80 had the compacted and smooth char. EDAX (energy dispersive spectroscopy) associated with SEM was used to get the elemental composition of residues of RPUF. Table 7 shows the surface composition of the residues obtained by EDAX. As can be observed from Table 7, C and O were the main remaining elements. The char layer of RPUF/P60 and RPUF/P80 can compress well to form a protective phosphatic barrier to resist the transfer of heat and mass during fire. As shown in Figure 12, a number of flaws were found on the surface of RPUF/Si60 and RPUF/Si80, which may be the result of SiO_2_ forming and moving onto the surface during burning. PTOP and PTOSi have the effect of promoting the char layers, as compacted char layers may prevent the internal products of pyrolysis from transferring to the flame zone.

### 3.7. Mechanical Property of RPUF

Mechanical property is an essential issue for the practical application of RPUF, which was further investigated, such as density, compressive strength and thermal conductivity. In order to eliminate the effect of RPUF density on the compressive strength, the specific compressive strength (compressive strength/density) was used to for comparison. 

Table 8 shows that the trend in RPUF density is opposite to the trend noted for compressive strength. With large amounts of PTOP and PTOSi added into the blended polyols component, the PTOP and PTOSi may disperse poorly, which can interfere with the development of the cell and make the cell size larger and the number of closed cells lower (Figure 5). PTOP can reduce the compressive strength of RPUF, while the higher content of PTOSi incorporated into RPUF can increase the compressive strength. A similar result was obtained for σ_sp_. When the PTOP content was below 60%, PTOP dispersed well in the blended polyols component, and almost no agglomeration appeared. PTOSi with its self-crosslinking function can make the RPUF matrix easily collapsible due to the wall fragility of the foam cell, resulting in the worse value of compressive strength.

RPUF is also used as the thermal insulation material in building insulation. The best method for improving the sustainability of building insulation is to diminish the thermal conductivity of RPUF. With an increase in the PTOP and PTOSi content, the thermal conductivity of RPUF decreased as the thermal conductivity is mainly affected by the average cell size. The thermal conductivity of RPUF/Si60 and RPUF/Si80 were higher than that of RPUF/P60 and RPUF/P80 due to the lower closed cell content, which is influenced by the degree of cross-linking, the equivalent weight of blended polyols and the content of various agents. Therefore, the molecular weight of polyurethane in RPUF/P60 and RPUF/P80 was lower than that for RPUF/Si60 and RPUF/Si80.

## 4. Conclusions

A novel method was developed to synthesize PTOP and PTOSi, which were proved to be effective flame-retardant polyols used in the formation of RPUF with excellent flame retardancy. Different PTOP and PTOSi contents affect the thermal stability, flame retardancy, and mechanical properties of RPUF. Results from FTIR, TG, DSC, and LOI revealed that DOPO and DPSD are linked to EGTO by a covalent bond, and the thermal stability of PTOP was higher than that for PTOSi. The thermal stability and flame retardancy of RPUF increased, with the LOI value of RPUFs with 80% PTOP and PTOSi reaching 24.0% and 23.4%, respectively. PTOP and PTOSi could be acted as the soft segment and hard segment in RPUF, respectively. The compressive strength of RPUF was reduced to 0.75 MPa with 80 wt % PTOP, while the compressive strength of RPUF increased to 0.25 MPa with 80 wt % PTOSi. A comparison of the flame-retardant mechanism of RPUF prepared by PTOP and PTOSi showed that RPUF with PTOP released an acid source and polyphosphate compound while RPUF with PTOSi burned out silicon dioxide on the surface. Based on the LOI value of RPUF, PTOP showed better flame-retardant performance than that of PTOSi.

## Figures and Tables

**Table 1:** The parameters of PTOP and PTOSi.

Sample	DOPO (mol)	EGTO/Epoxy Group (g/mol)	DPSD (mol)	Conversion Rate (%)	Viscosity (Pa·s)	P (%)	Si (%)	Hydroxyl Value (mg KOH/g)
PTOP	0.072	100/0.1875	0	95.85	2.19	1.85	0	339.96
PTOSi	0	100/0.1875	0.084	95.48	2.42	0	1.95	317.93

**Table 2:** Formulation of rigid polyurethane foam (RPUF).

Sample	Content Weight (g)							
	PPG4110	PTOP	PTOSi	AK8804	DBTDL	Water	HFC-365mfc	PAPI
Neat RPUF	100	0	0	2.4	1.2	1.2	20	120
RPUF/P60	40	60	0	2.4	1.2	1.2	20	120
RPUF/P80	20	80	0	2.4	1.2	1.2	20	120
RPUF/Si60	40	0	60	4.0	0.28	0.17	20	100
RPUF/Si80	20	0	80	4.0	0.11	0.11	20	100

**Table 3:** The parameters of TG and DTG of PTOP and PTOSi.

Sample	N_2_ Atmosphere		The Maximum Decomposition Rate (%/min)	Residue Rate (wt %)
	*T*_onset_ (°C)	*T*_max_ (°C)		
PTOP	272.40	468.10	11.31	4.13
PTOSi	228.20	440.10	6.18	8.58

**Table 4:** The parameters of TG and DTG of RPUF.

Sample	*T*_onset_ (°C)	*T*_max_ (°C)			Residue Rate (wt %)
		Step I	Step II	Step III	
Neat RPUF	313.10	354.30	478.10	/	17.54
RPUF/P60	282.60	330.90	408.70	461.40	20.48
RPUF/P80	261.80	319.60	402.30	460.60	19.20
RPUF/Si60	291.40	335.10	463.70	/	18.03
RPUF/Si80	288.50	338.60	464.50	/	17.00

**Table 5:** RPUF Limiting Oxygen Index (LOI) values.

Sample	Neat RPUF	RPUF/P60	RPUF/P80	RPUF/Si60	RPUF/Si80
LOI value (%)	19.0	21.3	24.0	21.0	23.4

**Table 6:** Cone calorimeter testing data for RPUF.

Sample	TII (s)	PHRR (kW·m^−2^)	Time to PHRR (s)	THR (MJ·m^−2^)
RPUF/P60	2	266.03	35, 75	22.78
RPUF/P80	3	259.38	40, 60	18.40
RPUF/Si60	2	285.36	30, 60	22.80
RPUF/Si80	3	255.20	40, 65	21.99

**Table 7:** EDAX data of the residue of RPUF.

Sample	C (wt %)	O (wt %)	P (wt %)	Si (wt %)	Others (wt %)
RPUF/P60	79.67	17.72	1.83	0	0.78
RPUF/P80	83.38	14.35	0.83	0	0.84
RPUF/Si60	77.37	20.61	0	1.13	0.89
RPUF/Si80	79.61	17.08	0	2.31	1.01

**Table 8:** Physical and mechanical properties of RPUF.

Sample	ρ (kg·m^−3^)	σ (MPa)	σ_sp_ (N.m.kg^−1^)	K (W·mk^−1^)
RPUF/P60	97.08 ± 0.44	0.82 ± 0.02	8446	0.039
RPUF/P80	89.48 ± 0.80	0.75 ± 0.03	8381	0.038
RPUF/Si60	57.09 ± 0.38	0.18 ± 0.02	3152	0.051
RPUF/Si80	52.94 ± 0.32	0.25 ± 0.02	4722	0.044

ρ: density; σ: compressive strength; σ_sp_: specific compressive strength; K: thermal conductivity.
